# Supplementary material for: Double-CRISPR Knockout Simulation (DKOsim): A Monte-Carlo randomization system to model cell growth behavior and infer the optimal library design for growth-based double knockout screens
Source: PLoS Comput Biol. 2026 Apr 17;22(4):e1013510. doi: 10.1371/journal.pcbi.1013510 (PMC13108905; doi:10.1371/journal.pcbi.1013510)
Supplement: S1 Text — (DOCX) [file pcbi.1013510.s001.docx]

**S1 Text. Supplemental Methods and Materials.**

**Connection between the Discrete and the Continuous Model.** In this subsection, we show the connection between the proposed discrete Multinoulli-based simulation model and the continuous exponential growth-based simulation model. The latter model assumes that the number of individuals at time $t$ is $N(t)=N_{0}exp(\kappa t)$ where $N_{0}$ is the number of individuals at time $0$ and $\kappa\in(-\infty,\infty)$ is the growth rate per unit of time (in our context a WT cell doubling) and $t$ is the time.

Under this continuous model at one unit of time (one unit of cell doubling), $t=1$, one cell with SKO on gene 1 will yield $exp(\kappa_{1})$ descendant cells; and similarly a cell with SKO at gene 2 will yield $exp(\kappa_{2})$ descendants; considering no interaction a cell with DKO will yield $exp(\kappa_{2}+\kappa_{1}-1)$ descendants. Next, we find the theoretical phenotype values of $p_{1}$ and $p_{2}$ generating an expected number of descendants under the proposed discrete model equal to the descendants, in one unit of time, under the continuous model.

With the model in (1) and (3), for one unit of cell doubling, we defined the following notations:

- Under SKO, the original cell yield descendants $C_{x_{1}}=1\left( x_{1}\neq0 \right)\times2^{x_{1}}\in\{0,2,4\}$
- Under DKO, the original cell yield descendants $C_{y}=1\left( y\neq0 \right)\times2^{y}\in\{0,2,4,8\}$

For SKO gene, given

$$x_{1}\sim Multinomial(n=1,values=\{0,1,2\}, prob=\{(-p_{1})_{+},1-|p_{1}|,(p_{1})_{+}\})$$

We consider the following cases for possible input of $p_{1}$:

1. $0\leq p_{1}\leq1$:

$$\begin{aligned} E(C_{x_{1}})&=2(1-|p_{1}|)+4(p_{1})_{+} \\ &=2(1-p_{1})+4(p_{1}) \\ &=2+2p_{1} \\ &=2^{\kappa_{1}} \\ 1+p_{1}&=2^{\kappa_{1}-1}\Rightarrow\boldsymbol{\kappa}_{\boldsymbol{1}}\mathbf{=lo}\mathbf{g}_{\mathbf{2}}\mathbf{(1+}\mathbf{p}_{\mathbf{1}}\mathbf{)+1} \end{aligned}$$

1. $-1\leq p_{1}<0$:

$$\begin{aligned} E(C_{x_{1}})&=0(-p_{1})+2(1+p_{1}) \\ &=2+2p_{1} \\ &=2^{\kappa_{1}} \\ 1+p_{1}&=2^{\kappa_{1}-1}\Rightarrow\boldsymbol{\kappa}_{\boldsymbol{1}}\mathbf{=lo}\mathbf{g}_{\mathbf{2}}\mathbf{(1+}\mathbf{p}_{\mathbf{1}}\mathbf{)+1} \end{aligned}$$

Without loss of generality, for SKO effects of gene 2, $\boldsymbol{\kappa}_{\boldsymbol{2}}\mathbf{=lo}\mathbf{g}_{\mathbf{2}}\mathbf{(1+}\mathbf{p}_{\mathbf{2}}\mathbf{)+1}$**.**

Thus, under the assumption of independence s.t. $x_{1}⟂x_{2}$, as in (3), given

$$\begin{aligned} y\sim Multinomial(n=1,&p_{0}^{y_{0}}=(-p_{1})_{+}(-p_{2})_{+}+(-p_{1})_{+}[1-|p_{2}|] \\ &+(-p_{1})_{+}(p_{2})_{+}+[1-|p_{1}|](-p_{2})_{+}+(p_{1})_{+}(-p_{2})_{+}, \\ &p_{1}^{y_{0}}=[1-|p_{1}|][1-|p_{2}|], \\ &p_{2}^{y_{0}}=[1-|p_{1}|](p_{2})_{+}+(p_{1})_{+}[1-|p_{2}|], \\ &p_{3}^{y_{0}}=(p_{1})_{+}(p_{2})_{+}) \end{aligned}$$

We consider the following three cases for all possible combinations of inputting $p_{1}$ and $p_{2}$:

1. $p_{1},p_{2}\geq0$:

$$\begin{aligned} E(C_{y})&=0\times p_{0}^{y_{0}}+2^{1}p_{1}^{y_{0}}+2^{2}p_{2}^{y_{0}}+2^{3}p_{3}^{y_{0}} \\ &=2\left\{ (1-p_{1})(1-p_{2})+2[(1-p_{1})p_{2}+p_{1}(1-p_{2})]+4p_{1}p_{2} \right\} \\ &=2\left\{ (1-p_{1}-p_{2}+p_{1}p_{2})+2[p_{1}+p_{2}-2p_{1}p_{2}]+4p_{1}p_{2} \right\} \\ &=2(1+p_{2}+p_{1}+p_{1}p_{2}) \\ &=2^{\kappa_{1}+\kappa_{2}-1} \end{aligned}$$

1. $p_{1},p_{2}\leq0$:

$$\begin{aligned} E(C_{y})&=0\times p_{0}^{y_{0}}+2^{1}p_{1}^{y_{0}}+2^{2}p_{2}^{y_{0}}+2^{3}p_{3}^{y_{0}} \\ &=2\left\{ (1+p_{1})(1+p_{2}) \right\} \\ &=2(1+p_{2}+p_{1}+p_{1}p_{2}) \\ &=2^{\kappa_{1}+\kappa_{2}-1} \end{aligned}$$

1. $p_{1}\geq0$, $p_{2}\leq0$:

$$\begin{aligned} E(C_{y})&=0\times p_{0}^{y_{0}}+2^{1}p_{1}^{y_{0}}+2^{2}p_{2}^{y_{0}}+2^{3}p_{3}^{y_{0}} \\ &=2\left\{ (1-p_{1})(1+p_{2})+2[(1-p_{1})\times0+p_{1}(1+p_{2})]+4p_{1}\times0 \right\} \\ &=2\left\{ (1-p_{1}+p_{2}-p_{1}p_{2})+2[p_{1}+p_{1}p_{2}] \right\} \\ &=2(1+p_{2}+p_{1}+p_{1}p_{2}) \\ &=2^{\kappa_{1}+\kappa_{2}-1} \end{aligned}$$

Therefore, we conclude that when $x_{1}⟂x_{2}$,

$$E(C_{y})=\frac{E(C_{x_{1}},C_{x_{2}})}{2}=\frac{E(C_{x_{1}})E(C_{x_{2}})}{2}=2^{\kappa_{1}+\kappa_{2}-1}$$

where

$$\left\{ \begin{matrix} \kappa_{1}=log_{2}(1+p_{1})+1 \\ \kappa_{2}=log_{2}(1+p_{2})+1 \end{matrix} \right.$$

This is, the expected number of descendant cells generated by one cell with DKO whose genes are not interacting after one unit of cell doubling is the same as the same cell under the continuous exponential growth model.

**Glossary of tunable components.**

1. $n=$ The number (#) of different genes: # of single target genes to be knocked out in both SKO and DKO, and combination pairs.
2. $C=$ The coverage of the experiment: # of cell representations per construct.
3. $n_{g}=$ # of guides per gene.
4. ${\%}_{GI}=$ The percentage (%) of genetic interactions: the % of interacting gene pairs among the gene pairs not containing a non-targeting control.
5. % of each gene type: for $k=1,\ldots,n$, we define
   - **Negative** (${\%}_{neg}$): $p_{k}<0$, a broader class of **essential genes**, genes whose KO results in detrimental effects towards the cell and lead to cell division **slower** than WT.
   - **Positive** (${\%}_{pos}$): $p_{k}>0$, genes whose KO results in benefiting the cell viability and lead to cell division **faster** than WT.
   - **Wild-Type (WT)** (${\%}_{wt}$): $p_{k}\approx0$, genes whose KO unaltered cell viability and cell will divide normally, **presents variance**.
   - **Non-targeting Control (CTRL)** (${\%}_{ctrl}$): $p_{k}=0$, functioning the same as WT, but flagged for statistical analysis later, strictly equal to 0 **without variance**.

- *Note: It is assumed that Non-targeting controls do not interact with any other genes.*

1. $\sigma_{f}$= standard deviation of the log 10 of the initial frequency distribution of the SKO gene
2. ${\%}_{heg}=$ % of high-efficacy guides
3. Cell Doubling Time via Bottleneck specification:
   - $n_{b}=$size: a threshold that indicates the ceiling of cell growth. Once the colony exceeds this number, it reaches a bottleneck. At this point, the colony is split to reduce the cell number.
   - $n_{e}=$ # of encounters: # of times that the colony of cells will be allowed to pass the threshold $n_{b}$ and then reduced by splitting it.

- *Note: Both* $n_{b}$ *and* $n_{e}$ *are designed to control the cell doubling time. To simplify the computational complexity, we set* $n_{b}$ *to an integer that indicates how many times it is relative to the initial library size, and users can increase the cell doubling time by increasing either* $n_{b}$ *or* $n_{e}$ *as desired to implement more simulated cell passage procedures.*

1. $\lambda=Multiplicity of infection \left( moi \right)$: the % of cells that are transfected by any virus, built upon the following assumptions :
   - Follows a Poisson process during transfection
   - $\lambda<0.5$ and only select cells with single transfection occurrence:

$$P(x=1;Poisson(\lambda))$$

Based on users’ inputs, the following cell library parameters are further calculated:

1. $n_{c}=$ # of unique constructs

$$:=\frac{n\times n_{g}\times(n-1)\times n_{g}}{2}+n\times n_{g}$$

For example, if $n_{g}=2$, two guides are targeting each gene. Denoting $a$ and $b$ as the two guides targeting gene 1, gene 1 yields two constructs $1,a$ and $1,b$. Denoting $c$ and $d$ as the guides targeting gene 2, the gene pair 1;2 yields 4 constructs: (1) $1,a;2,c$, (2) $1,a;2,d$, (3) $1,b;2,c$ and (4) $1,b;2,d$. The “,” separates the gene from the guide targeting it, and the “;” separates the genes. The first term in the expression above is the number of constructs with 2 genes, while the second term is the number of constructs with one gene.
2. $L_{0}=$Initial library size: # of cells before transfection at baseline.

$$:=n_{c}\times C$$

$C$ is the average number of cells per construct.
3. $n_{comb}=$# of combinatorial genes for both SKO and DKO.

$$=:\frac{n\times(n-1)}{2}+n$$

4. $n_{r}=$Resampling Size: # of cells to sample (without replacement) once a bottleneck has been reached.

$$=:Poisson(\lambda)\times n_{b}$$

5. $C_{pseudo}=$pseudo-count: a constant added to the relative frequency of the constructs at both the initial timepoint $t_{0}$ and later timepoint $t_{2}$ to avoid $-\infty$ when calculating log fold change

$$=:5\times{10}^{-\lfloor\log_{10}(n_{b})\rfloor-1}$$

$\lfloor a\rfloor$ is the integer less than or equal to $a$.

**Simulation System Design II: Cell Population Transfection and Selection**

**Independent Viral Transduction and Replications.** Typically, in CRISPR experiments, the biological experimental replicates start in library cloning and lentivirus production[1], or after the lentiviral library infection[2]. Building upon the assumptions of independent viral transduction in the cell library, we treated the initialized *S*KO *and DKO* genes as the plasmid (pDNA) in the simulation model with guide-efficacy designs. As such, two independent sets of the initialized cell library containing both KO genes and targeting guides following the methods specified in the *Guides Initialization* Section are constructed for cell population transduction, transfection, and selection, where each set of the initialized library is named as replicate A (repA) and replicate B (repB), respectively. Two simulated replications, **RepA** and **RepB,** are set at the stage of cell library initialization: for each simulation run, given the same sets of initialized $SKO(k)$ and $DKO(k_{1};k_{2})$, we set up two cell libraries with guides:

1. Cell library repA containing $SKO_{A}(k,j)$ and $DKO_{A}(k_{1},j_{1};k_{2},j_{2})$
2. Cell library repB containing $SKO_{B}(k,j)$ and $DKO_{B}(k_{1},j_{1};k_{2},j_{2})$

We implemented independent cell population transfection and selection on both. We mainly calculated Pearson’s Correlation $r$ between the relative frequency of the constructs and the LFC by replicates at each timepoint to measure the reproducibility of the simulated experiments.

The primary purpose of designing two replicates was to demonstrate the reproducibility in our CRISPR experimental simulations, and to align with practical laboratory designs that often incorporate at least two biological replicates for a complete screening.

#### **LFC Z-Standardization.** To compare the LFC on the same scales across different simulated designs, based on $L_{guide}^{2}$, we aggregated LFC from (16) in both replicates by means. Additionally, by adopting ideas from LFC Z-score[3] and gene Zscore[4]), for each construct $i$, we incorporated the z-standardized Log2 Fold-Change (zLFC) at $t_{2}$ vs. $t_{0}$ by

$$zLFC_{i}=\frac{LFC_{i}-\mu_{control}}{\sigma_{control}}$$

where $\mu_{\text{control}}$ and $\sigma_{\text{control}}$ are the mean and standard deviation of the LFC values among the constructs in the control group (*i.e.*, the control group contains sets of $SKO(k,j)$ and $DKO(k_{1},j_{1};k_{2},j_{2})$ where $k,k_{1},k_{2}$ are all Non-Targeting Controls), given by the following:

$$\mu_{\text{control}}=\frac{1}{R}\sum_{r=1}^{R} \text{LFC}_{r}, \sigma_{\text{control}}=\sqrt{\frac{1}{R-1}\sum_{r=1}^{R} \left( \text{LFC}_{r}-\mu_{\text{control}} \right)^{2}}$$

where $r\in\text{control group}$ and $R$ is the total number of unique constructs in the control group.

#### **Experimental Procedure Tracking.** To track the transfection and selection progress throughout the simulation stages for iterations and bottleneck encounters, we created a log files to dynamically collect all information during the sampling and cell growth procedure, including the following entities: simulation sample name, replication, timestamp of starting and completing execution, counters of iterations (cell doubling cycles), and counters of encountered bottlenecks.

### **Simulation System Design III: Optimization and Utility**

To reduce the computational cost of the simulation, we further optimized our schematic designs of the system by vectorization and parallel computing.

### **Optimization.** To fully accelerate the simulations, we optimized the cell library construction, cell population transfection, and selections as follows:

1. Vectorized the row-wise processing of the cell library
2. Defined the matrix $M_{p}$ that stores the cell division probabilities ${\mathbf{p}_{\mathbf{y}}}^{'}:=({p_{0}^{y}}^{'},{p_{1}^{y}}^{'},{p_{2}^{y}}^{'},{p_{3}^{y}}^{'})$
3. Vectorized the cell counts computation for each construct from multinouli growth functions by (4) along the sequence of the cell library entities with its corresponding $\mathbf{p}_{\mathbf{y}^{\mathbf{'}}}$ in $M_{p}$

### **Utility.** For utility designs, we wrapped up the methods and simulations into four main functions as follows:

1. initialize_gene_cell_lib0(): Cell Library Initialization on $SKO\left( k \right)$, $DKO\left( k_{1};k_{2} \right)$ and $I_{k_{1},k_{2}}$.
2. initialize_guide_cell_lib0(): Cell Library Initialization on $SKO\left( k,j \right)$, $DKO\left( k_{1},j_{1};k_{2},j_{2} \right)$, $\boldsymbol{f}_{\boldsymbol{C}_{\boldsymbol{0}}}^{\boldsymbol{rel}}$, $c_{0}^{g_{k,j}}$, and $c_{0}^{g_{k_{1},j_{1};k_{2},j_{2}}}$.
3. define_phenotype_gi(): Cell Division Probability Vectors Calculations on $\mathbf{p}_{\mathbf{y}}^{\mathbf{0}}$, $\mathbf{p}_{\mathbf{y}}, {\mathbf{p}_{\mathbf{y}}}^{'}, \pi.$
4. run_replicate(): Cell Population Transfection and Selection Simulation on two replicates.

We practically ran the simulations by utilizing the written functions in standard stepwise pipelines.

### **Parallel Computing in High-Performance Computing.** We additionally employed parallel computing to utilize the high-performance computing cluster resources, as shown below:

1. Parallel cell library initialization on two replicates using initialize_gene_cell_lib0().
2. Parallel computation of $\mathbf{p}_{\mathbf{y}}^{\mathbf{0}}$, $\mathbf{p}_{\mathbf{y}}, {\mathbf{p}_{\mathbf{y}}}^{'}, \pi.$ using define_phenotype_gi().
3. Parallel simulation of cell population transfection and selection process in two replicate cell libraries using run_replicate().

For this, we requested 40 cores with 256G RAM memory, with a total runtime of 240 hours allotted for the parallel computing nodes. The runtime is tracked in units of hours, and we collected it by the end of each simulation.

### **Algorithmic Designs: Monte-Carlo Simulation on Large Scales**

#### **Simulation Steps.** We summarized the practical computational workflow of our simulation design to DKOsim on large scales as follows (**S5 Fig**):

1. Set up initial parameters
2. Defined main functions
3. Initialized the theoretical phenotypes for the 1st and 2nd target genes $p_{k_{1}}$ and $p_{k_{2}}$, correspondingly
4. Initialized the interaction index $I_{k_{1},k_{2}}$ among all gene pairs from pre-specified genes
5. Initialized $SKO(k)$ and $DKO(k_{1};k_{2})$
6. Initialized guides that target the 1st and 2nd genes $j_{1}$ and $j_{2}$ with type and efficiency
7. Initialized the cell library with constructs containing all pre-specified genes and guides $SKO(k,j)$ and $DKO(k_{1},j_{1};k_{2},j_{2})$
8. Calculated cell division probability vectors $\mathbf{p}_{\mathbf{y}}^{\mathbf{0}},\mathbf{p}_{\mathbf{y}},{\mathbf{p}_{\mathbf{y}}}^{'}$ and genetic interaction $\pi$
9. Defined the cell population transfection and selection, including cell growth by ${\mathbf{p}_{\mathbf{y}}}^{'}$ and sub-sampling from a hypergeometric distribution using equation (14)
10. Utilized compiled functions to run simulations in two replicates to store the simulated data

**References**

1. Shen JP, Zhao D, Sasik R, Luebeck J, Birmingham A, Bojorquez-Gomez A, et al. Combinatorial CRISPR–Cas9 screens for de novo mapping of genetic interactions. Nat Methods. 2017;14: 573–576. doi:10.1038/nmeth.4225

2. Hart T, Chandrashekhar M, Aregger M, Steinhart Z, Brown KR, MacLeod G, et al. TKO: High-Resolution CRISPR Screens Reveal Fitness Genes and Genotype-Specific Cancer Liabilities. Cell. 2015;163: 1515–1526. doi:10.1016/j.cell.2015.11.015

3. Shifrut E, Carnevale J, Tobin V, Roth TL, Woo JM, Bui CT, et al. Genome-wide CRISPR Screens in Primary Human T Cells Reveal Key Regulators of Immune Function. Cell. 2018;175: 1958-1971.e15. doi:10.1016/j.cell.2018.10.024

4. Colic M, Wang G, Zimmermann M, Mascall K, McLaughlin M, Bertolet L, et al. DrugZ: Identifying chemogenetic interactions from CRISPR screens with drugZ. Genome Med. 2019;11: 52. doi:10.1186/s13073-019-0665-3
