# Supplementary material for: Double-CRISPR Knockout Simulation (DKOsim): A Monte-Carlo randomization system to model cell growth behavior and infer the optimal library design for growth-based double knockout screens
Source: PLoS Comput Biol. 2026 Apr 17;22(4):e1013510. doi: 10.1371/journal.pcbi.1013510 (PMC13108905; doi:10.1371/journal.pcbi.1013510)
Supplement: S1 Table — (DOCX) [file pcbi.1013510.s004.docx]

**S1 Table. Toy example: Single Gene KO parameters.**

| Row number | $k$ | Gene class | $p_{k}$ | $f_{0}^{'k}$ |
| --- | --- | --- | --- | --- |
| 1 | 1 | Negative | -0.40 | 2.42 |
| 2 | 2 | Wild type | -0.03 | 0.80 |
| 3 | 3 | Control | 0.00 | 2.54 |
