# Supplementary material for: Double-CRISPR Knockout Simulation (DKOsim): A Monte-Carlo randomization system to model cell growth behavior and infer the optimal library design for growth-based double knockout screens
Source: PLoS Comput Biol. 2026 Apr 17;22(4):e1013510. doi: 10.1371/journal.pcbi.1013510 (PMC13108905; doi:10.1371/journal.pcbi.1013510)
Supplement: S2 Table — (DOCX) [file pcbi.1013510.s005.docx]

**S2 Table. Toy example: Single & Double KO Genes Initialization.** Rows above (below) the dashed lines represent single (double) KO genes. Genes 1 and 2 interact and, for example, $f_{k_{1},k_{2}}$ for the DKO of genes 1 and 2 is the mean of $f_{0}^{'k_{1}}$ and $f_{0}^{'k_{2}}$ of these genes, i.e., $1.61=\frac{2.42+0.8}{2}$. In rows 5 and 6, 1st(2nd)-gene $p^{I}$ equals 1st(2nd) gene $p$ because there is no interaction $I=0$. The second column denotes the values of $k$ for SKO and $k_{1}$ for DKO.

| row | 1st gene | 2nd gene | 1st gene | 2nd gene | interaction | 1st gene | 2nd gene | $f_{k}$ |
| --- | --- | --- | --- | --- | --- | --- | --- | --- |
|  | $k$ or $k_{1}$ | $k_{2}$ | $p_{k_{1}}$ | $p_{k_{2}}$ | flag ($I_{k_{1},k_{2}}$) | $p_{k_{1}}^{I}$ | $p_{k_{2}}^{I}$ | or $f_{k_{1},k_{2}}$ |
| 1 | 1 |  | -0.40 |  |  |  |  | 2.42 |
| 2 | 2 |  | -0.03 |  |  |  |  | 0.80 |
| 3 | 3 |  | 0.00 |  |  |  |  | 2.54 |
| 4 | 1 | 2 | -0.40 | -0.03 | 1 | -0.27 | 0.01 | 1.61 |
| 5 | 1 | 3 | -0.40 | 0.00 | 0 | -0.40 | 0.00 | 2.48 |
| 6 | 2 | 3 | -0.03 | 0.00 | 0 | -0.03 | 0.00 | 1.67 |
