# Supplementary figures and images for: Double-CRISPR Knockout Simulation (DKOsim): A Monte-Carlo randomization system to model cell growth behavior and infer the optimal library design for growth-based double knockout screens
Source: PLoS Comput Biol. 2026 Apr 17;22(4):e1013510. doi: 10.1371/journal.pcbi.1013510 (PMC13108905; doi:10.1371/journal.pcbi.1013510)

**S3 Table. Toy Example: Initial Cell Library.**


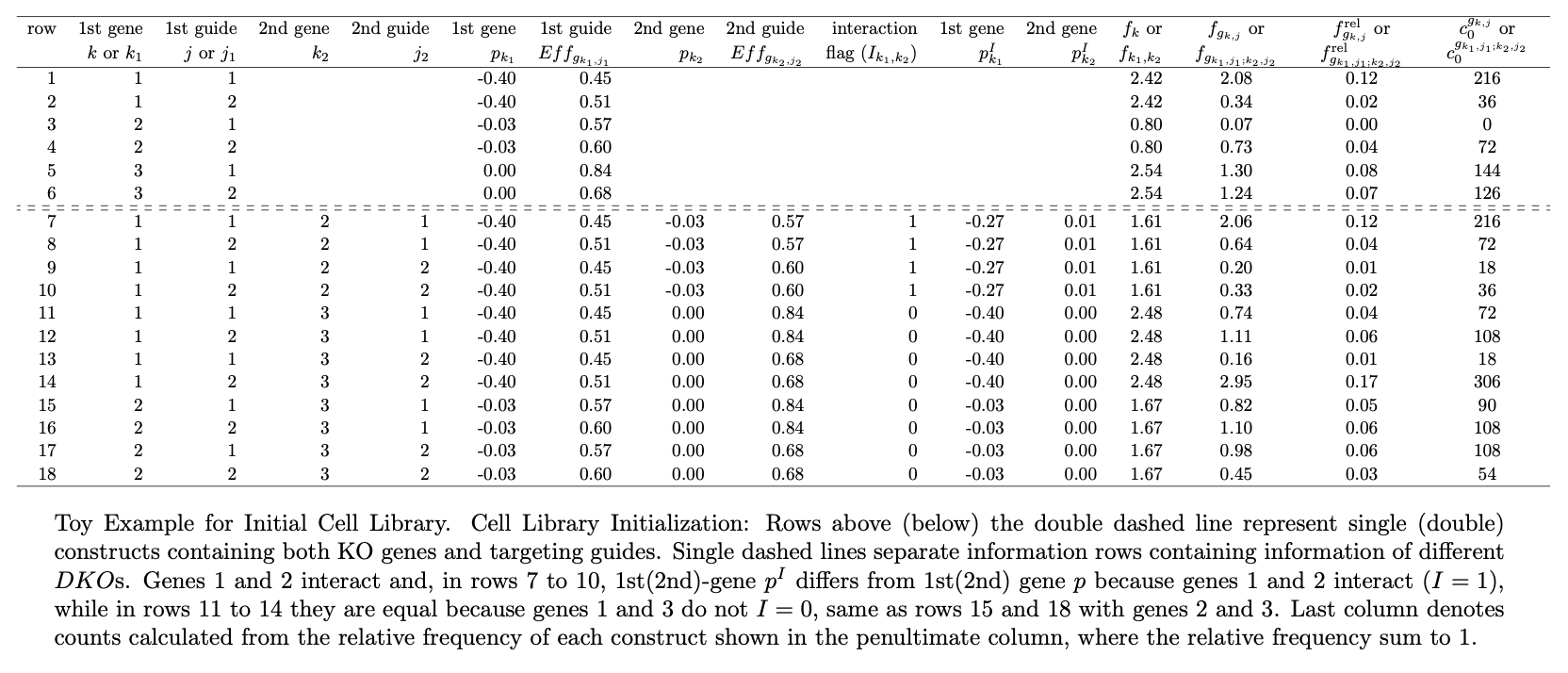

Supplement: S3 Table — (DOCX) [file pcbi.1013510.s006.docx]
