# Supplementary material for: Double-CRISPR Knockout Simulation (DKOsim): A Monte-Carlo randomization system to model cell growth behavior and infer the optimal library design for growth-based double knockout screens
Source: PLoS Comput Biol. 2026 Apr 17;22(4):e1013510. doi: 10.1371/journal.pcbi.1013510 (PMC13108905; doi:10.1371/journal.pcbi.1013510)
Supplement: S4 Table — (DOCX) [file pcbi.1013510.s007.docx]

**S4 Table. Summary Table for Fong 2024 Data Characteristics.**

| **Fong et al. (2024)[23]: SCHEMATIC, Combinatorial CRISPR-Cas9** | |
| --- | --- |
| **# of cell lines** | 7 tumor cell lines: MDAMB231, A549, A427,  CAL33, CAL27, MCF7, MCF10A |
| **Initial cell library size** | 300,000 in each well of a 6-well plate |
| **Coverage of library production** | *>* 100× |
| **# of targeted single genes** | 67(frequently mutated genes)  176(druggable genes) |
| **# of targeted gene pairs** | 11792 = 67 x 176  176(druggable genes) |
| **# of guides (gRNAs) per gene** | 3 |
| **# of cell representation per guide** | > 500 |
| **Multiplicity of infection (MOI)** | 0.3 |
| **# of dual guide RNA constructs (dgRNAs)** | 110,728 with inclusion of negative controls |
| **# of timepoints** | 4 |
| **# of replicates** | 2 |
