# Supplementary material for: Double-CRISPR Knockout Simulation (DKOsim): A Monte-Carlo randomization system to model cell growth behavior and infer the optimal library design for growth-based double knockout screens
Source: PLoS Comput Biol. 2026 Apr 17;22(4):e1013510. doi: 10.1371/journal.pcbi.1013510 (PMC13108905; doi:10.1371/journal.pcbi.1013510)
Supplement: Attachment — Submitted filename: Response to Reviewers.docx [file pcbi.1013510.s011.docx]

**Response to reviewers' comments:**

Key: reviewer comments in red; responses in black; "changes to text quoted in light blue"

**Comments from Reviewers:**

**Reviewer #1 (Comments to the Author):**

Summary

In summary, this paper presents the first double gene knockout (DKO) simulation framework for CRISPR screening in modeling cell growth behavior using mathematical modeling and Monte Carlo simulation. The overall idea is good and the model design is sound. It is very promising and important to extend from single gene knockout (SKO) simulation to double knockout (DKO) modeling.

In general, the introduction and methods are well-written, with clear toy examples that help readers understand the meaning of each parameter and experimental condition. The model itself is also clear and captures the biological process, especially the dynamics of cell growth.

However, in the results section, I have several specific suggestions. Overall, I understand the author’s logic — first demonstrating that the model is tunable, then showing that it can accurately approximate experimental data, and finally confirming that the results are reproducible. Below are my detailed comments for each part.

1. Evaluation of parameter influence on model performance

1.1 This section validates the influence of each parameter on model performance using AUC-PR and Pearson correlation. Overall, the evaluation metrics are appropriate, and the results clearly show performance trends with respect to parameter changes. Including the BAGEL method for comparison is also a good choice.

However, I do not think this analysis should appear as the first result — it might fit better later in the results section.

Reply: We appreciate the reviewer for this suggestion. In the results section we have three aims:

(1) to show that the simulated data behaves as we would expect in terms of its relationship of the model parameters and the correlations between the simulation truth and the estimated interactions; for example we expect the correlation to increase when coverage, percentage of high efficacy guides and the number of guides and to decrease when the standard deviation (dispersion) of the initial counts increase;

(2) to show that simulation scheme can produce real lab data patterns;

(3) to show the reproducibility of simulation. The figure of this section appears in the order of the aims.

It is important to present the influence of each parameter as the first result to show that the effect of each designed tunable parameter in the simulation is behaving as expected, so that we could move towards further validation.

1.2 Moreover, I suggest evaluating the joint effects of multiple hyperparameters (e.g., changing coverage and another parameter simultaneously) to test whether there are synergistic effects among them.

Reply: We appreciate the reviewer for this suggestion. Currently, we have chosen six parameters to compare the simulation tunability, including coverages, percentage of high-efficacy guides, GI magnitude, dispersion of the initial frequency of SKO counts, number of guides per gene, and cell doublings. To evaluate the joint effects of multiple parameters, we additionally test the synergistic effects with more simulation runs as follows:

(1) between coverage (at 10x, 100x, and 200x) and percentage of high-efficacy guides (from 10%, 30%, 50%, 80% to 100%), visualized in Fig. 4D-F;

(2) between coverage (at 10x, 100x, and 200x) and dispersion of the initial frequency of SKO counts (from $\frac{1}{1.05}$, $\frac{1}{1.35}$, $\frac{1}{1.68}$, $\frac{1}{2.07}$, $\frac{1}{2.56}$, to $\frac{1}{3.29}$), visualized in Fig. 4G-I.

Visualizations to the results are added to the line diagrams in Fig.4:

We explicitly refined our interpretations and described these new results on the test of synergistic effects in the text –

“We then compared the tunability for the percentage of high-efficacy guides. When the systematic runs were restricted to compare guide quality by tuning the high-efficacy guides percentages at 100x coverage, the correlations between dLFC and simulated GIs with increasing percentage of high-efficacy guides from 10% to 100% showed an increasing trend, with r = 0.76 for 100% high-efficacy guides (**Fig. 4D**). We additionally tested whether there are synergistic effects between high-efficacy guides percentage and coverage. Simulations were run on increasing percentage of high-efficacy guides from 10% to 100%, stratified by coverage at 10x, 100x, and 200x. Results showed that correlations increase with higher coverage overall, comparing the same level of percentage of high-efficacy guides. We observed the effect of increasing percentage of high-efficacy guides on correlation is synergistically enhanced by higher coverage, particularly at low-to-moderate efficacy ranges, where improvements in guide-efficacy translate into disproportionately larger gains in correlation.

**Fig. 4E** shows BAGEL essentiality identification results: while BAGEL AUC-PR in recovering SKO gene essentiality is high (AUCPR > 0.75) across almost all conditions, benefits of increasing high-efficacy guides percentage depend on coverage, with substantial improvements at low coverage and diminishing returns once coverage is sufficient. This suggests an interaction but weak-additive synergy interaction between high-efficacy guides percentage and coverage for SKO gene essentiality identification. To evaluate GI identification performance from dLFC, we examined changes in Precision/Recall@80 for the top-ranked negative genetic interactions across increasing percentage of high-efficacy guides (Fig. 4F), as well as AUC–PR for all negative GIs (S2 Fig. B). Both precision and recall increased with guide efficacy; however, the magnitude of improvement strongly depended on library coverage. Specifically, increases in the proportion of high-efficacy guides yielded the largest gains in both Precision@80 and Recall@80 under low-coverage conditions at 10x, whereas performance improvements diminished as coverage increased to 100x and 200x. At moderate to high coverage, Precision@80 approached 0.95, indicating that approximately 76 of the top 80 ranked interactions correspond to true simulated negative GIs. In contrast, recall improved more gradually and exhibited earlier saturation, reflecting the greater difficulty of exhaustively recovering all true interactions. Together, these results demonstrate a coverage-dependent, synergistic interaction between library coverage and guide efficacy, whereby improvements in guide efficacy disproportionately enhance GI detection performance when guide sampling is limited, with diminishing returns once coverage becomes sufficient.

To demonstrate the tunability of the initial counts dispersion, following **Table 1**, we systematically ran DKOsim by tuning $\sigma_{f}$ based on the z-score resulting from a 40-90% confidence level. For example, at 90% confidence, the expected z-score is 3.29, and we constructed the SKO gene frequency following a normal distribution with $\sigma_{f}=\frac{1}{3.29}$ so that there is a 10-fold difference between the 95^th^ and 5^th^ percentiles. As defined, with 100x coverage, lower confidence leads to higher $\sigma_{f}$, resulting in a higher dispersion of the initial count distribution. Based on this setting, we visualized the changes of the correlation r with increasing setting to the dispersion of initial frequencies of SKO counts (**Fig. 4G**). The increments of the initial counts dispersion decrease the correlation r on dLFC vs. simulated GI overall, and we found that r drops to 0.58 when $\sigma_{f}=\frac{1}{1.05}$. On metrics of correlation r, we tested whether there are synergistic effects between the initial counts dispersion and coverage. Increasing dispersion in initial SKO counts’ frequencies consistently reduced the correlation between dLFC and simulated GIs; however, the magnitude of this effect depended on library coverage. In particular, high dispersion caused a disproportionate loss of correlation under low-coverage conditions, whereas higher coverage partially buffered against uneven starting frequencies. These results indicate a synergistic interaction between library coverage and the initial SKO counts dispersion, whereby limited sampling and skewed guide abundances jointly amplify stochastic noise and degrade GI detection performance from dLFC.

We applied BAGEL essentiality identification on simulations in this setting. Increasing dispersion in initial SKO counts’ frequencies had only a modest effect on SKO gene essentiality identification performance, with AUC-PR remaining high (AUCPR > 0.75) across most conditions (Fig. 4H). While higher dispersion led to gradual performance degradation at low to moderate coverage, high coverage largely buffered against uneven starting frequencies, maintaining near-ceiling AUC-PR values. Unlike GI detection, SKO essentiality exhibited a saturating response to both coverage and initial frequency dispersion, indicating limited interaction and no strong synergistic effect between these parameters. Increasing dispersion in initial SKO counts’ frequencies led to a pronounced decline in both Precision@80 and Recall@80 for top negative GI detection (Fig. 4I). Importantly, the magnitude of this degradation depended strongly on library coverage: under low-coverage conditions, even moderate dispersion caused a rapid collapse in both precision and recall, whereas higher coverage substantially mitigated these effects. The highly non-parallel performance trajectories indicate a strong synergistic interaction between coverage and initial frequency dispersion, whereby limited sampling and skewed guide abundances jointly amplify stochastic noise and severely compromise GI detection.”

We also want to highlight that Fig. 4J is already including the implicit test of synergistic effect between the number of guides per gene and the percentage of high-efficacy guides, and we addressed the review’s comments related to this in query#1.3.

1.3 I also have some questions about the results shown in Fig. 1D–F and Fig. 1J. In Fig. 1D–F, PCC increases monotonically with the proportion of high-efficiency guides, but in Fig. 1J, the PCC is not highest when the rate of high-efficiency guides reaches 100%.

Reply: We appreciate the reviewer for catching these unexpected patterns. We run the simulation multiple times to collect the response, and the response stays the same as shown in Fig. 4J. But as we mentioned in the manuscript, dLFC might not identify genetic interaction (GI) well in a perfect guide-efficacy scenario and choosing 3 guides per gene would be enough in real laboratory screening. Multiple literatures (Doench et al., 2016; Hart et al., 2017; Sanson et al., 2018) also support that an empirical range of 50% to 100% is reasonable for modern CRISPR guide efficacy, and selecting 3 guides would be sufficient in most library designs, and that is the primary objective to be presented here.

1.4 I wonder why this occurs — perhaps the authors could perform simulation experiments to analyze the relationship between high-efficiency guide rate and number of guides per gene, rather than only relying on PCC.

Reply: We appreciate the reviewer for posting this question. The relationship between high-efficiency guide rate and number of guides per gene is reflected in Fig. 4J, as we have performed multiple simulation experiments to evaluate the joint effects between these two hyperparameters. But since there might be some concerns, we explicitly added the description to interpret the relationship between high-efficiency guide rate and number of guides per gene in the text –

“Our simulation reflected the synergistic effects between high-efficacy guide rates and the number of guides per gene in CRISPR screens. Considering a correlation of at least 0.75 for GI to be effectively identified, for sets of the simulation with high-efficacy guides proportions lower than 50%, more guides for each target gene (≥ 5) need to be incorporated into library design. For experiments with higher proportions of high-efficacy guides (≥ 80%), two guides are generally sufficient. This shows that the number of guides per gene and high-efficacy guide proportion interact synergistically, with more guides set for each target yielding disproportionately greater stability when higher fractions of guides achieve strong knockout efficiency.”

1.5 Finally, I recommend providing a summary or guideline for hyperparameter selection at the end of this section. There are many tunable parameters (around six), and even though the model is adjustable, users would benefit from practical reference values or examples for parameter tuning.

Reply: We agree with the reviewer that providing a summary for hyperparameter selection would be beneficial for users. We have prepared an R package named DKOsimR for running DKO simulation. A default hyperparameter used in the function has been described in the vignettes of the R package accessible at https://github.com/yuegu-phd/DKOsimR. We have added a section on **Summary Guidance in Picking Suitable Parameters** in the tutorial files of the built R package. We also added a direction to our prepared summary of guidelines to pick parameters by the end of this section in the text –

“We prepared a summary of guidance on parameter selection for running simulations. The guideline can be accessed in “Summary Guidance in Picking Suitable Parameters” section from the tutorial in vignettes of DKOsimR, our built R package for DKOsim. Check Data Availability Statement to install and access DKOsimR.”

2. Model approximation to real data

2.1 This section evaluates how well the model approximates real experimental data by analyzing data variance (s). However, it is unclear why the authors did not calculate and visualize s in the same way as in Fig. 5F, which would make it easier to directly compare whether the simulation perfectly approximates the experimental data.

Reply: We appreciate the reviewer pointing out the confusion caused by using s. The primary focus is on the histograms, which show, visually, that the log10 normal distribution assumed by our model, and by CRISPulator, for the initial library counts can be used to approximate the initial distribution of the real dataset. Once the initial counts are similar, along with other specific parameter values, the cell growth is generated visually similar to the real dataset. Since S_lab can take the reader’s attention off the main point above, we removed it.

2.2 The meaning of s (data variance) also needs more explanation — specifically, how it is used to measure approximation quality and why it is a suitable metric. This explanation could be added before the results or in the supplementary materials, with a clear citation in the results part. Otherwise, the role of s is confusing to readers.

Reply: We thank the reviewer for this suggestion. Please see our reply to query#2.1 to address this related issue. s is the sample variance from the data and we will remove it to minimize the distractions to the main points.

2.3 Additionally, I think the authors should include a baseline method for comparison. Even though DKOsim may be the first tool for DKO simulation, it would still be informative to slightly modify an existing SKO simulation method and compare it against DKOsim. This would better demonstrate the model’s relative performance and contribution.

Reply: We sincerely appreciate the reviewer for this suggestion. While we also wish we could compare a baseline model with our designed DKOsim, it is unfortunately not feasible for several primary reasons:

1. As the reviewer mentioned, DKOsim is the first tool for DKO simulation that considers the combinatorial CRISPR screening design. The goal of DKOsim is to simulate gene-gene interactions for comparing different GI identification methods and optimizing future DKO experiments. All other existing simulation tools do not contain simulated GI effects, thereby making a comparison of simulated GI to other existing simulation tools impossible.
2. Most of other existing CRISPR simulation is built for FACS screens, and we mainly designed a simulation framework for growth-based screens. Since FACS screens simulations do not model cell population changes over time, and the detection methods have fundamental differences between the two, it would not be possible to compare DKOsim to FACS-based simulation.
3. We adapted the Monte Carlo methods from CRISPulator (Nagy et al., 2017) and extended it from SKO to DKO design; part of our methodology refers to CRISPulator, and you may have observed how we develop an existing SKO simulation method into DKO design. However, the tutorial of CRISPulator is mainly for FACS-based screens, and as mentioned, we think it is not comparable with DKO in growth-based screening design. To our knowledge, CRISPulator is the only CRISPR simulation tool that considers growth-based design at this time.

3. Reproducibility and biological validation

3.1 The third part focuses on the model’s reproducibility and its consistency with real data. However, I find the first part of the experiment somewhat repetitive with Fig. 4 or the first results section. Perhaps the authors could use alternative visualization methods to express these results more clearly.

Reply: We appreciate the reviewer’s comments in catching this confusion and agree that the first part of the reproducibility might be found somewhat repetitive with Fig. 4. However, in our current simulation design, each simulation run is inherently consisting of two replicates, named replicate A and replicate B, and the essence of model’s reproducibility part in Fig. 6 is to visualize the correlation between these inherent replicates, and compare with the correlation from replicates in actual laboratory data. To be more specific, Fig. 6 A-C is visualizing the calculated correlations between replicate A and replicate B within each simulation run; while the correlation from Fig. 4 is the correlation between the dLFC identified gene-gene interaction with the simulated truth. The sets of correlation being compared are different between the two results sections. Since we also need to show the dynamics of the simulated CRISPR screening results, Fig. 6 A-C would be the best way to reflect the dynamics of the simulation results, considering one of the parameters is being tuned by future users.

3.2 Since the simulation involves randomness, I also suggest performing the experiments multiple times and adding error bars to show the variability.

Reply: We sincerely thank the reviewer for this suggestion. Please see our replies to query#3.1 above. As mentioned, each simulation run is inherently consisting of 2 independent replicates. This simulated design is set to mimic independent viral transduction from actual CRISPR screens and we have explicitly described this design in subsection **Independent Viral Transduction and Replications** from **S1 Text. Supplemental Methods and Materials.** As written in the text –

“As such, two independent sets of the initialized cell library containing both KO genes and targeting guides following the methods specified in the *Guides Initialization* Section are constructed for cell population transduction, transfection, and selection, where each set of the initialized library is named as replicate A (repA) and replicate B (repB), respectively. Two simulated replications, **RepA** and **RepB,** are set at the stage of cell library initialization: for each simulation run, given the same sets of initialized $SKO(k)$ and $DKO(k_{1};k_{2})$, we set up two cell libraries with guides:

1. Cell library repA containing $SKO_{A}(k,j)$ and $DKO_{A}(k_{1},j_{1};k_{2},j_{2})$
2. Cell library repB containing $SKO_{B}(k,j)$ and $DKO_{B}(k_{1},j_{1};k_{2},j_{2})$

We implemented independent cell population transfection and selection on both. We mainly calculated Pearson’s Correlation $r$ between the relative frequency of the constructs and the LFC by replicates at each timepoint to measure the reproducibility of the simulated experiments.”

Since each simulation contains 2 replicates, each point on the lines from the line diagrams in Fig. 6A-C is a result of correlation after performing the experiment twice. The variability across simulation experiments with different parameters is visualized by connecting each point to a line.

3.3 The second part of this section presents a good idea, but the validation is currently too limited — relying only on Pearson correlation is insufficient. The authors could further analyze specific examples from both simulated and real datasets, combined with biological background or known gene interactions, to provide stronger validation and insights.

Reply: We sincerely appreciate the reviewer’s comments and suggestions. We agree that relying only on Pearson correlation is insufficient to compare simulated and real datasets. We mainly compare the simulation and real experimental data in the second results section, where we have shown the high overlap between simulation and real data in terms of distributions of initial counts, log fold change after simulated cell growth, and genetic interactions.

The major finding in this section is to emphasize the correlation between the two inherent replicates of each simulation resembles the correlation between replicates from actual laboratory data across two different lentiviral designs. We tried to research some alternative metrics for comparing the reproducibility between simulated and real datasets. One of the best choices we found is to incorporate a within-vs-between context replicate correlation (WBC) score (Billmann et al., 2023). Unfortunately, we found that DKOsim at the current stage might not incorporate different contexts, and it is focusing more on its methods of tunability and reproducibility for this study. And since Pearson correlation r is widely used as the most popular metric in real lab screening analysis, we think PCC would still be the most appropriate choice for comparison. A combined comparison between simulation and real data would be our next step in future work. We hope the reviewer understands our concerns at this time. We have reflected this as a limitation in our text of discussion –

“Third, we mainly rely on Pearson correlation coefficient to validate DKOsim’s reproducibility but this might not consider context difference when comparing to real experimental data. Future comparisons on context-specific replicates need to take involved for a through comparison between simulations and real datasets.”

3.4 Finally, it would be valuable if the authors could discuss the gene-gene interactions captured in the simulation and compare them with those observed in experimental data, which would highlight the biological relevance of their model.

Reply: We thank the reviewer for this suggestion. A direct comparison of the gene-gene interactions between the simulation and experimental data has been included and shown in Fig. 5E, where the interactions captured in the simulation are highly overlapping with those observed from real lab data. These results have been described to highlight that our designed double knockout simulation framework is highly relevant to biology in recapitulating the gene-gene interaction patterns in actual CRISPR screens.

4. Additional concerns and suggestions

4.1 In the end, I still have several concerns about the work.

First, I am not fully convinced by the validation based solely on Pearson correlation (PCC). It seems insufficient to demonstrate that the simulated data are of high quality. I strongly recommend including specific case examples comparing simulated and real datasets, which would make the validation more intuitive and convincing.

Reply: We strongly agree with the reviewer’s comment that the inclusion of examples comparing simulation and real lab data is important for validation. And a direct comparison between simulated results and real datasets is currently included in Fig. 5, where we compare the distribution between simulation and laboratory data in terms of initial constructs’ counts, log fold change, and genetic interaction. Case examples included in the figures are also demonstrated in the second major component of the results, and we showed that the constructed simulation can be utilized to approximate patterns from actual laboratory double-CRISPR knockout screening data. These comparisons are not just on Pearson correlation, but also on the shapes of all mentioned distributions and variances between the two.

4.2 Second, although I found a Jupyter notebook on GitHub, there is no clear user documentation explaining how to use this simulation method. The provided analysis_example notebook is quite difficult to follow — the cell_lib section, in particular, is excessively long and affects readability. It might be sufficient to include only a concise example of the cell library instead. In the discussion section, the authors claim that the model is user-friendly, even for users with limited background, but without readable tutorials and proper documentation, this claim is difficult to verify.

Reply: We appreciate the reviewer for pointing out the difficulty in using the model. Please see our reply to query#1.5, we have written a complete R package named DKOsimR that is installable for all future users now. The prepared R package is accessible and installable at https://github.com/yuegu-phd/DKOsimR, and we also provide a vignette with a detailed tutorial to practically use the package to run the simulation in a simple way. Now that the R package consists of all the codes and models described in the text, we believe the model is user-friendly, and users with a limited background but with some basic knowledge of using R can run simulations to generate synthetic CRISPR screening data that resemble patterns from actual lab screens.

4.3 Third, I hope to see more insightful biological or practical analyses beyond Pearson correlation, precision, and recall. For instance, it would be interesting to explore whether models trained on this DKO simulated dataset could improve downstream tasks, such as gene expression perturbation prediction, compared with models trained on SKO simulated datasets. Currently, the first set of results feels somewhat preliminary and may be more appropriate as supplementary material.

Reply: We sincerely appreciate the reviewer for raising these directions of exploration. We strongly agree with the reviewers' expectation that these DKO simulated datasets will improve downstream tasks. However, the scope of the current study is primarily on designing and constructing a DKO simulation system that is tunable and applicable to resemble data from actual laboratory CRISPR screens on DNA. We believe the asymptotes we found by running simulations are helping investigators to optimize the variables that are most critical to generate data from actual screens. The method itself is also a major component of the current studies that have been validated for both tunability and reproducibility. We are currently working on writing a manuscript that benchmarks several genetic interaction identification methods based on simulations, and seeking ways to develop the current interaction identification methods. Gene expression perturbation prediction on RNA is an interesting direction, but we think it would be future work beyond the current study. And since there are no other simulation tools for DKO screens, the comparison to other DKO simulation models trained on SKO simulation would be impossible to our knowledge at the current stage.

4.4 Finally, I have questions regarding parameter selection, particularly the threshold separating high- and low-efficiency guides at 0.6. Why was 0.6 chosen? Is there any experimental evidence supporting this threshold?

Reply: We sincerely appreciate the reviewer for bringing this question to our attention. There is no experimental evidence supporting the 0.6 threshold. Nevertheless, in our designed simulation framework, both mean efficiency of high and low-efficacy guides $\mu_{high}$ and $\mu_{low}$ are tunable by users, with default values of 0.9 (sd=0.1) and 0.05 (sd=0.07). By default, the guide efficacy for both high and low categories drawn from randomization almost never reaches 0.6. Although the chance of overlap is negligible, this threshold is used to ensure that the overlapping probability is exactly zero, thereby guaranteeing that the high-efficacy guides always have higher efficacy than the low-efficacy guides. So the choice of 0.6 is not changing anything; it is mainly for clear separation between high and low-efficacy from the guides. We explicitly discussed this in the main text –

“There is no experimental evidence supporting the threshold of 0.6. Nevertheless, both mean efficiency of high and low-efficacy guides $\mu_{high}$ and $\mu_{low}$is tunable by users, with default values 0.9 ($\sigma_{high}$=0.1), and 0.05 ($\sigma_{low}$=0.07). The guide efficacy for both high and low categories drawn from randomization almost never reaches 0.6 by default. This threshold is used to ensure that the overlapping probability is exactly zero, thereby guaranteeing that the high-efficacy guides always have higher efficacy than the low-efficacy guides.”

4.5 Moreover, while the model is tunable, it remains unclear how users should determine suitable parameter combinations in practice. Are these parameters estimated based on real data characteristics or by another optimization strategy? Providing such guidance would greatly improve the model’s usability.

Reply: We strongly agree with the reviewer that providing a guidance on determining suitable parameter combinations in practice would improve current model’s usability. In the vignettes of our built R package for DKO simulation, we added the “Summary Guidance in picking suitable parameters” as Section 7, to provide a general suggestion for future users to pick hyperparameters. This guidance lists our picks of parameters used from empirical DKO simulation runs that are efficient for generating simulated datasets. Please see our reply to query#1.5, where we added a direction to our prepared summary of parameter picking strategies in the text by the end of the first section of results, which points to our vignettes files for using DKOsimR. We also attach the link to our pre-built vignettes that can be accessed at https://github.com/yuegu-phd/DKOsimR/blob/main/inst/extdata/vignettes/DKOsimR.pdf.

We believe this summary guidance will help future users and investigators adjust their parameter choices based on different desires and purposes when running DKO simulations.

**Reviewer #2 (Comments to the Author):**

Summary

1. The authors designed a computational framework to simulate single KO and double KO fitness effects typical for CRISPR screening experiments. Double KO simulation can be used to study and optimize the effects of library parameters in order to obtain optimal gene interaction data from screenings. While the general concept of the paper has its merits and the authors have identified an interesting gap to be addressed, the study itself has weaknesses that can not be ignored: The figures presented in the paper are barely legible due to low resolution and overload of text.

Reply: We appreciate the reviewer bringing this critical visibility issue to our attention and apologize for the inappropriate figure formatting that impairs readability. We have converted all included figures to formats using PLOS’s NAAS tool. All figures presented in the paper have been processed by NAAS with confirmation that they meet the requirements for PLOS publications. They should now be legible with appropriate resolution and print size.

2. The computational frame work is a single R script, that is poorly documented and not according to the standard of today's scientific software development, and the actual usability for other researchers is questionable due to the specific assumptions that the authors make.

Reply: We sincerely appreciate the reviewer for bringing this code documentation issue to our attention. Please see our reply to query#1.5 of Reviewer 1 to a related issue. We have packaged all R scripts into an R package named DKOsimR, which is downloadable and installable by future users. All documents related to the written R package are stored in GitHub page on <https://github.com/yuegu-phd/DKOsimR>. We have also provided a tutorial document inside the vignettes of the R package to help users get started with the functions we wrote.

Major comments:

3. - Motivation for the study: the authors state that the two methods to identify genetic interactions (CTG, Gemini) that they used resulted in almost random GI for the original experimental test data, and that this motivated the need for a simulated data set. Why this motivation is understandable, it feels weak because there might be other methods around to calculate genetic interactions, and if not, it should rather motivate the authors to investigate why these methods failed to show good overlap.

Reply: We thank the reviewer for this suggestion and agree that including only two genetic interaction methods is limited to fully support the motivation for developing a simulation framework. While we also include dLFC as another genetic interaction detection method to validate the simulation in the later sections of the results, we are currently preparing the manuscript to compare and benchmark other GI detection methods, together with CTG and GEMINI. Unfortunately, since the study in this manuscript focuses on constructing the DKO simulation itself, we primarily demonstrate the simulation methods and validate them in terms of tunability and reproducibility at the current stage.

4. To use simulated data somehow feels as if the authors try to evade the complexity of real experiments. This leads also to one of the fundamental criticism for this manuscript: how useful is simulated data really, in comparison to real experimental data?

Reply: We sincerely appreciate the reviewer for bringing this critical question to our attention. We design a double CRISPR knockout simulation mainly for the purpose of inferring an optimal library design of real DKO experiments. The essential method of the current simulation framework is Monte-Carlo randomization, where we derived the probability for possible outcomes of cell division with targeted knockout genes. The method is based on some biological assumptions adapted from CRISPulator (Nagy et al., 2017) to simplify the mathematical derivation steps, which we have stated in the methods section of the text. To answer the reviewer’s question, we are not evading the complexity of real experiments, but rather connecting the statistical rigor from Monte-Carlo methods to approximate the real laboratory data pattern for the most essential part of the real experiments.

We agree with the reviewer that showing how useful the simulated data in comparison to real experimental data is critical, and as in the results sections of our manuscript, the second aim is to include a direct comparison between the simulated dataset and the real experimental data pattern. This is visualized in Fig. 5, where the simulated data are highly overlapping with real experimental data in terms of distributions for both initial counts, log fold change, and genetic interactions. Furthermore, in our final aim of the results, we compared the reproducibility between simulated and experimental data, and we observed that sets of correlations between replicated simulated data can resemble experimental data in two different lentiviral delivery designs. All these results validate that our simulated data can be utilized to approximate real experimental data, and the designed Monte-Carlo simulation method is converging to real lab settings. The simulation design is derived from the essential components of real biological CRISPR screens.

Methods:

5. "in SKO cells, we assumed a single-guide (sgRNA) disrupts one gene, whereas in DKO screens, two distinct guides were combined as one dual-guide (dgRNA) to perturb two genes simultaneously"

The situation in real experiments is often much more complex: one guide can have quite variable binding, knock-down or knock-out efficiency, which is why real CRISPR library experiments often feature up to 5 guides and more for target a single gene. This makes it questionable how well simulated data can recapitulate actual library experiments.

Reply: We thank the reviewer for this comment. We agree that the situation in real experiments is often more complex, where the guide efficacy effects have to be taken into consideration in the library design. To address this issue, we incorporate the design of knockout guide-efficacy in our simulation model, where the theoretical knockout effects from the initialized target genes would be affected by guides with variable knockout efficiency. Based upon the incorporation of guide-efficacy design, we compare the simulated data with actual experimental data. Results are visualized in Fig. 5 where we showed that simulated data can recapitulate actual library experimental data in distributions of initial counts, log fold change, and genetic interactions.

6. "knockout of gene 1 will yield, in terms of cell division, one of the following

three outcomes, in one unit of WT cell doubling time: (a) 1 = 0: Cell does not divide and loses viability; (b) 1 = 1: Cell divides once as WT; (c) 1 = 2: Cell divides twice."

A discrete model where cells do divide not at all, equal to WT, or twice as much as WT seems highly unrealistic. From published studies and own work I know that fitness effects are most often subtle changes in growth rate, but still measurable over time, but never 2x WT growth rate.

Reply: We sincerely appreciate the reviewer for sharing insights and comments from his own work to bring this critical question to greater attention. We agree that the growth rate of WT cells never reaches 2x, and our current simulation methods do not contradict this fact. In our design, the growth rate of cells is derived from the theoretical phenotype of the target gene $p_{1}$. Considering cells with the highest growth rate in our design (i.e., cells with positive target KO), a default value for $p_{1}$ is drawn from a truncated normal distribution ranging from 0.025 to 1 with mean 0.75 and variance 0.01, where $p_{1}$ is almost never reaching 1, and this implies ${1+p}_{1}<2$ in math. So the case $p_{1}=1$ is extremely rare and almost never happens, but since we are adapting Monte-Carlo simulation method to construct the framework, all of the possible events have to be considered to fully capture the dynamics of cell growth with knockout genes. But still, the growth rate of cells never reaches 2x growth rate of WT cells.

7. The authors do seem to allow fine-tuning of guide efficacy as stated in Table 1, but it is not clear how this connects with dicsrete model assumption.

Reply: We apologize for not stating the connection clearly enough, which may have confused the reviewer during their reading. In our current methodology design, guide-efficacy lower than 100% reduces the cell growth below the induced cell growth by theoretical phenotype $p_{1}$. The theoretical phenotype $p_{1}$ and $p_{2}$ that would be associated with the cell growth if the efficacy were 100% as shown in equations (1) and (2) for SKO, and in equation (8) for DKO. Cell growth on a SKO or DKO depends on the theoretical phenotypes $p_{1}$ and $p_{2}$ that determine the growth when guide-efficacy $Eff_{g_{k,j}}=1$. The efficacy is connected to the model by equation (13) where it reduces the values of $p_{1}$ and $p_{2}$ used by the model, and the growth of cells with KO genes would be multiplied by guide-efficacy. For example, if efficacy is zero, $p_{1}^{'}=0$ and the cell behaves like WT. We have added a sentence explaining this in the text –

“More specifically, by (1), (2) and (8), cell growth on a SKO or DKO depends on theoretical phenotypes $p_{1}$, $p_{2}$ that determine the growth when $Eff_{g_{k,j}}=1$. Equation (13) above decreases the cell growth rate of cells infected with low-efficacy guides compared to the cells infected with high-efficacy guides.”

8. One foot note reads "3. We are referring to the mean and sd of normal distribution before being truncated to generate the theoretical phenotypes of each class of genes" which points towards transformation of continuous input to the discrete model.

Reply: We appreciate the reviewer pointing out this confusion, which requires further clarification. The foot note is clarifying that we are parameterizing the truncated normal distribution in terms of the mean *µ* and sd *σ* of the non-truncated version; the mean *µ* and sd *σ* are different from the mean and sd of the truncated version. This distribution is just to generate the theoretical phenotype $p_{1}$ and $p_{2}$ that would be used to generate the discrete (counts) model.

Thus, the model is discrete by construction at the level of cell division dynamics, while continuous fitness parameters govern the probabilities of these discrete division outcomes. Observed discrete readouts (counts) then arise from these probabilistic division processes and subsequent sampling steps, rather than from an explicit continuous-to-discrete transformation of theoretical phenotypes.

To connect our discrete model to the continuous case, we have written a section in **S1 Text. Supplemental Methods and Materials**, named “**Connection between the Discrete and the Continuous Model**” to show the connection between the two, where the effects of SKO gene is derived in continuous case of $\kappa_{1}$ and $\kappa_{2}$. We have explicitly refined our description to this connection in the text –

“In this subsection, we show the connection between the proposed discrete Multinoulli-based simulation model and the continuous exponential growth-based simulation model. The latter model assumes that the number of individuals at time $t$ is $N(t)=N_{0}exp(\kappa t)$ where $N_{0}$ is the number of individuals at time $0$ and $\kappa\in(-\infty,\infty)$ is the growth rate per unit of time (in our context a WT cell doubling) and $t$ is the time.

Under this continuous model at one unit of time (one unit of cell doubling), $t=1$, one cell with SKO on gene 1 will yield $exp(\kappa_{1})$ descendant cells; and similarly a cell with SKO at gene 2 will yield $exp(\kappa_{2})$ descendants; considering no interaction a cell with DKO will yield $exp(\kappa_{2}+\kappa_{1}-1)$ descendants. Next, we find the theoretical phenotype values of $p_{1}$ and $p_{2}$ generating an expected number of descendants under the proposed discrete model equal to the descendants, in one unit of time, under the continuous model.

(mathematical derivations are skipped here)

This is, the expected number of descendant cells generated by one cell with DKO whose genes are not interacting after one unit of cell doubling is the same as the same cell under the continuous exponential growth model.”

9. "For every ( 1 ; 2 ) an interaction indicator 1 , 2 was generated. Within the set of DKOs not containing a non-targeting control, we randomly selected % of DKOs and flagged their genes as interacting (coded as = 1) and the rest as not interacting ( = 0)."

There seems to be now way to control which set of genes can be reproducibly flagged as GI-positive or negative, only the percentage. Re-running the same function will then lead to different results every time, due to the random sampling steps? This feels not very reproducible for experimentalists that e.g. want to test small toy data sets, but is probably OK for large data sets.

Reply: We thank the reviewer for bring this reproducibility issue to our attention and we agree that the reproducibility for small toy datasets need to be ensured. To solve the issue, we have added a tunable “rseed” variable in R function used to run simulations, where users can assign same values of random seed to each simulation run to ensure reproducibility is attained for both small toy data and large datasets. We have tested two small toy datasets by setting same “rseed”, the same sets of gene pairs are reproducibly flagged to have GI, with same simulated GI values. This use of “rseed” is further clarified in the vignettes (tutorial) file for the R package, where it is defined as a value used for random number generator; users may input a same number to control the same sets of genes reproducibly having GI.

Results:

10. - Figure 3 is only text; what is the purpose of this figure? It reiterates the input parameters from table 1 and the mathematical description of the algorithm presented in another chart. This leaves the impression that the authors want to inflate the presented results, which is merely a single R script at the moment.

Reply: We sincerely thank the reviewer for pointing out this confusion. The main purpose of Figure 3 is to provide readers an overview of the design and analytical frameworks of our study. While we agree with the reviewer that the input parameters overlap with the terms in Table 1, but with a brief summary of each input parameter, we emphasize a general workflow to help readers understand what the core essentials of the simulation model are and what the primary outputs should be expected from users’ inputs. Since the methodology in the text describes all mathematical derivations, we feel it is tedious for readers to go through all details. Therefore, we include this overview diagram to provide a high-level summary that helps readers understand the workflow of our study. And all input, model, output, and analytical workflow are covered in the methods and results section after Figure 3 is presented, with a prepared R package; we think this figure does not inflate any part of the results, but enhances the readability for more future users of our designed simulation framework.

11. - Figure 4: not readable at all due to low resolution. Include vector graphics instead of pixel graphics into the manuscript.

Reply: We thank the reviewer for this suggestion and apologize for the formatting issue. Please see our reply to query#1. We have converted all figures presented in the manuscript with PLOS’s NAAS tool to a TIFF file that meets all requirements for publication. All figures included have passed the requirements check from PLOS’s NAAS tool, which should resolve the readability issue.

12. The line diagrams in the figure show dependence on 1 variable, e.g. coverage, but it is not clear how many simulations were run when changing this parameter, like, was coverage simulated in steps of 10%, 1%, 0.1%, and why is there no variation visible?

Reply: We appreciate the reviewer for pointing out this confusion. Each line in the diagrams plots the calculated correlation between dLFC and the simulated genetic interaction truth, while keeping all other variables constant to visualize the tunability for one specific parameter. The simulations were run by tuning parameters in steps, and each point corresponds to one simulation output. For example, for coverage in Fig. 4A, simulations were run for coverage as 1, 5, 10, 30, 50, 80, 100, 300, 500x (this is a total of 9 simulations), and we analyzed each simulated data to calculate a Pearson correlation between dLFC and simulated GI truth. We then connect the calculated correlation to generate the line diagrams. Since each point on the line corresponds to one simulation run, there is no variation in the lines. We have added a clarification in first results section in the text –

“One simulation was run for each unique combination of parameters, and we visualized the analytical outputs in line diagrams (Fig. 4).”

13. The curves seem to change abruptly in some of the subfigures (B, E, F, ...), indicating rather sparse simulations? If not please explain the appearance of step-changes, or run more simulations.

Reply: We thank the reviewer for bringing this question to our attention. To explain the step changes in the subfigures, for example, Fig. 4B includes AUC-PR from BAGEL to identify the simulated negative gene calculated from 9 different simulations (same coverage level as in our reply to query#12). At 5xThe increment to BAGEL AUC-PR itself follows a step change pattern at 5x and 100x coverage. The goal of presenting the plot is mainly to show that BAGEL, as one of the most widely used method for gene essentiality identification, can be applied to our simulation and yield promising results with 0.99 AUC-PR at 100x. Similarly, in Fig. 4E and F, we plot the results from simulations with a percentage of high-efficacy guides at 10%, 30%, 50%, 80%, and 100%, and hold all other parameters constant, ensuring the comparison is interpretable. Results from BAGEL and dLFC precision/recall are showing a step change pattern, but overall, the tunability and applicability of our designed simulation framework has been shown.

14. - The simulation of library parameters in Figure 4 is actually really interesting and insightful. Only the poor presentation spoils the utility of these results.

Reply: We thank the reviewer for bringing this presentation issue to our attention. Please see our replies to queries#1 and #11 in addressing a similar readability issue. We have used PLOS’s NAAS tool to convert all figures presented, and this should solve the presentation issue in the results section.

15. - The provided software repository contains mainly a single R script with some documentation added directly to the script. Nowadays it should not be asked too much if software is provided and maintained in a more robust and user-friendly format.

Reply: We agree with the reviewer that the current simulation framework should be written into an R package instead of just a single R script for the ease of future users and investigators. Related to this issue, please see our reply to query#2. We have prepared an R package named DKOsimR that is freely downloadable and installable at <https://github.com/yuegu-phd/DKOsimR>. We also prepared vignette files as a tutorial for users to utilize the package in running their own simulation of the built-in R package. To further enhance the robustness of the package, we created a Release v1.0.0 and future updates would be directly recorded in the release history. In this way, we believe the double CRISPR knockout simulation framework we have constructed is now more robust and user-friendly.

16. Why is it not released as an R package? Building R packages is straight-forward and it could be released on CRAN or Bioconductor (better because more specific to biology).

Reply: We sincerely appreciate the reviewer for this suggestion. Please refer to our replies in the related queries#2 and #15. We strongly agree that an R package should be prepared and we have prepared and released one named DKOsimR. Following the tutorial documents, we hope this R package is straight-forward to use now.

17. While the math and algorithms seem to be correct, essential elements of scientific code are missing, such as: proper README in mark down; proper LICENSE, packaging as mentioned, with proper version and releases; avoiding hard-coded problematic parameters, e.g. the script uses 100% of all available CPUs without asking (!); modularization, import of functions from sub-scripts; test data and test routines (probably present as additional variants of the script?); user-defined (not hard-coded) output files and paths, ...

Reply: We sincerely appreciate the reviewer for these suggestions. We have added a proper README in mark down file on the built R package; a proper MIT license has been created where LICENSE file is included; the DKOsimR R package has been assigned version 1.0.0 and released on Github for future use; we avoid the use of all available CPUs for parallel computing by setting 1 free cores by default, and number of free cores can be adjusted by users; we added subscripts to import functions running simulation in different modes; test data, routines, and saving path are all set to be tunable by users, and users now can define output files and paths following the tutorial documentations we provided.

18. - The authors seem to focus on eukaryotic model organisms such as human cell lines. This is OK but limits the usability of this highly specific framework even more. The authors should consider to make parameters like MOI (viral infection) or passages (cell lines) optional, or replace with more general parameters that can be used for bacteria too. Many CRISPR screenings are performed in bacteria (probably more than in human cells), and it feels unnecessary to exclude this audience simply by poor design.

Reply: We sincerely thank the reviewer for these thorough and valuable suggestions. In our current design, both MOI, the number of passages, and the size of the bottleneck to conduct passage are tunable. By default, we set MOI to be 0.3, with one time of passage, and passaging the cells when the cell population size reaches twice of initial library size. Within both R functions dkosim() and dkosim_lab() in our built DKOsimR package, we have made all these parameters tunable. Based on users’ desires and purpose in future use, both moi and passages are optional to be adjusted.

We agree with the reviewer’s suggestions that cell-line heterogeneity needs to be considered in the current framework, and our current simulation output is mainly validated through human cancer cell line. Unfortunately, tuning specifically to some commonly used cell lines, which are not limited to human but also include bacterial ones, would be future work that is beyond our current scope of study. However, since we have designed the parameters for both MOI and passages to be tunable, future investigators may increase MOI and adjust passages to adapt our simulation framework for simulating actual CRISPR screens performed in bacteria.

19. - All in all the manuscript is probably too long and too verbose for the limited scope of the presented results. The authors could try to condense the text and focus on the main findings (related to Figure 4). One example from the results section: "Building upon the tunability of DKOsim, we utilized the designed systems to approximate the real laboratory Double-CRISPR Knockout screening data, demonstrating the approachable feasibility of our simulation design in mimicking the CRISPR experiments in real-world settings". This is non-sense, just state the facts in plain English: "We compared the experimental data to the predictions made by our simulation".

Reply: We sincerely appreciate the reviewer’s suggestion with a clear example on how to better state the facts. We have replaced this pointed sentence with a more plain language in the text –

“We compared the actual experimental data to the synthetic data approximated by our simulation.”

20. There are many sections where the results are very verbosely described, which makes it harder (at least in my personal opinion) to extract the main messages.

Reply: We thank the reviewer for this suggestion and agree that many sections are verbosely described. Please refer to our reply in query#1.1 of Reviewer 1, we mainly presented three aims in our results to validate DKOsim:

(1) to show that the simulated data behaves as we would expect in terms of its relationship of the model parameters and the correlations between the simulation truth and the estimated interactions;

(2) to show that simulation scheme can produce real lab data patterns;

(3) to show the reproducibility of simulation.

To help readers extract the main messages with less difficulty, we reduce many redundant descriptions to details of the results. For aim 1, please refer to our reply in query#1.2 of Reviewer 1. We added tests for synergistic effects and used more plain language to explain the overall trend from the simulation results, shifting the focus from detailed observations for each parameter.

For aim 2, please refer to our reply in queries#2.1 and #2.2 of Reviewer 1. We removed the comparison of s for its distraction to major findings of this section, and we also removed all detailed descriptions of s, and decreased the use of redundant articles such as “the”, “a” in the text to ensure main messages could be conveyed to readers more smoothly. We also used more plain English to convey main idea from the study, for example, we replaced the original sentence on “Based on the aligned distributions of the constructs’ counts at the initial timepoint, we studied the feasibility of our designed system in simulating the cell growth, transfection, and selection that could eventually approximate the expected functional genomic data pattern from laboratory screenings.” by text –

“Based on the aligned distributions of constructs’ counts at the initial timepoint, we investigated whether DKOsim could simulate actual cell growth and approximate real experimental data patterns on LFC.”

For aim 3, we removed several verbose words for descriptions, and tried to keep the results centralized on reproducibility after presenting the first two aims. One example of refining text is –

“DKOsim is reproducible to maintain both stochastic consistency across Monte-Carlo randomizations and a sense that users across many disciplines can obtain consistent simulation results.”

For discussion, we also removed several redundant articles “the”, and refined the text with the updated DKOsimR package –

“DKOsim emphasizes user-level reproducibility: the platform provides a modular simulation R package named DKOsimR, ensuring future researchers from diverse backgrounds can reliably reproduce results by following the same defined workflow and tutorials.”

We hope reviewers and readers can extract main messages from our study more smoothly now.
